# Nonsuicidal self-injury as the gateway and consequence of suicidal ideation among adolescents: a cross-lagged regression analysis

**DOI:** 10.3389/fpsyt.2024.1434191

**Published:** 2024-08-15

**Authors:** Zhansheng Xu, Nianqin Li, Yaxin Kong, Lin Lin, Yu Liu, Huan Zhang, Yunfeng He, Song Zhao

**Affiliations:** ^1^ Key Research Base of Humanities and Social Sciences of the Ministry of Education, Academy of Psychology and Behavior, Tianjin Normal University, Tianjin, China; ^2^ Faculty of Psychology, Tianjin Normal University, Tianjin, China; ^3^ Center of Collaborative Innovation for Assessment and Promotion of Mental Health, Tianjin, China; ^4^ Intelligent Laboratory of Child and Adolescent Mental Health and Crisis Intervention of Zhejiang Province, Zhejiang Normal University, Jinhua, Zhejiang, China; ^5^ Department of Psychology, Zhejiang Normal University, Jinhua, Zhejiang, China; ^6^ Liaoning Key Laboratory of Psychological Testing and Behavior Analysis, Liaoning University, Shenyang, China; ^7^ Liao Ning University Judicial Authentication Center, Liaoning University, Shenyang, China

**Keywords:** nonsuicidal self-injury, suicidal ideation, Chinese adolescents, cross-lagged regression, longitudinal study

## Abstract

**Background:**

There is a consensus that both nonsuicidal self-injury (NSSI) and suicidal ideation as risk factors for suicidal behavior have a strong connection. However, a lack of longitudinal information has limited the clarification of the concrete relationship between them.

**Aims:**

This study aimed to examine the specific mechanism between NSSI and suicidal ideation over time, during adolescence.

**Method:**

A longitudinal study was conducted with 193 Chinese adolescents. NSSI and suicidal ideations were examined over the course of a 1year followed-up, and three waves of data were collected.

**Results:**

The NSSI at time T1 significantly positively predicted suicidal ideation at time T2; Suicidal ideation at time T2 also significantly positively predicted NSSI at time T3.

**Limitations:**

Given that the small number of participants with suicidal ideation and NSSI, the findings of the study should be interpreted with caution and a lager sample is needed in the future.

**Conclusion:**

It was suggested that NSSI may occur before suicidal ideation, which in turn would strengthen NSSI, so interventions should be carried out from two aspects (behaviors and thoughts) to improve adolescents’ mental health.

## Introduction

*NonSuicidal Self-Injury (NSSI)* refers to a series of behaviors that directly, intentionally, and repeatedly harm one’s own body without suicidal intention, including cutting, scratching, burning, and other intentional destruction of body tissues, which is not socially acceptable (1, 2). The destructive consequences of NSSI make it extremely dangerous (3). For instance, NSSI is one of the most important risk factors for suicide (4–6). Although the purpose of self-injury is not suicide, individuals who engage in NSSI are much more likely to commit suicide or suicide attempts than others (7–10). A longitudinal study found that the risk of suicide in the first year was 0.7% in NSSI, which is 66 times higher than in the general population and the risk of suicide increased to 3% fifteen years later (11). Previous research also finds that NSSI and suicide are closely linked since 10% to 37% of NSSI patients attempt suicide at some point in their lives (12, 13). As a key stage of individual development, it is very important to pay attention to the issue of adolescents’ NSSI.

A large amount of evidence has confirmed that adolescents are at high risk of NSSI (4, 14, 15). Adolescents may have more vulnerability to be impulsive and with less mental resilience in the state of psychological and physiological immaturity (16). They cannot properly manage their negative emotions and some of them are prone to performing some extreme NSSI behaviors (17, 18). The incidence of NSSI was approximately 4% in the general population (19) and was much more common (13.9% ~ 40.2%) in adolescents (20, 21). As a major public health concern, the rate of NSSI in adolescents is on the rise in both China and Western countries (22, 23). Meanwhile, the occurrence of NSSI varies by region. The prevalence of NSSI among Chinese adolescents (31.4%~57.4%) is higher than that in Western countries (2, 14). NSSI is a repetitive behavior, as 55.1% of the group who engaged in the behavior at baseline continued to partake in NSSI in the following 6 months, while the proportion of adolescents seeking assistance in preventing was very small (24).

There was no doubt that NSSI and suicidal ideation are closely related. Studies have shown that the presence and frequency of NSSI can predict suicidal ideation (25, 26). Regarding NSSI itself, studies have shown that individuals who engaged in self-injury are more likely to report simultaneous suicidal ideation with an odds ratio of 8.39 (27). Research in a sample of 1,561 adolescents aged 14–24 years also suggested who engaging in NSSI had a high risk for suicidal ideation and some of its affecting factors, such as depression (28). To date, most studies focused on suicidal ideation and NSSI as co-occurrence variables (29), but there was little research on the time dimension mechanism between them. NSSI was seen in some studies as one of the most robust predictors of both suicidal thoughts and behaviors (30, 31); however, it seems less solid as a predictor in other studies (32, 33). A recent study using the ecological momentary assessment (EMA) to monitor a sample of borderline personality disorder patients for 7 days found that NSSI reduced suicidal ideation within the next few hours (34). Thus, the confusion lies not only in whether NSSI increases or decreases suicidal ideation, but also the near absence of longitudinal research considering the role of suicidal ideation in NSSI. It is also unclear to consider only the relationship between the two, because previous studies have confused suicidal ideation and behavior, as well as other mental disorders.

Given that there is currently no agreed-upon theory to explain the relationship between adolescents’ NSSI and suicidal ideation, we can only hypothesize the link between them with some existing theories. The interpersonal psychological theory of suicide (IPTS) emphasized NSSI can be considered as an indispensable “gateway or tool” for individuals to improve their acquired ability for engaging suicide (35, 36). An integrated model, consistent with IPTS, is used to understand acquired capability for suicide will at least partially mediate the link between NSSI and suicidal behavior (30). Neuroimaging evidence showed that the pain processing of self-injured individuals was abnormal, and pain could be seen as a reward to explain their repeated addictive NSSI (37). Several studies have demonstrated that individuals with a repeated history of NSSI exhibit greater pain tolerance and pain thresholds (i.e., less fear of pain) (38–40). A questionnaire survey of inpatient psychiatric sample proved that NSSI can predict suicide readiness by increasing individual pain tolerance and fearless of death (41). The above evidence showed that if NSSI and suicidal behavior exist as a continuous process, then NSSI should be considered as the development before suicidal behavior (10, 12). Based on the IPTS, the present study hypothesized that NSSI would predict suicidal ideation later.

How the NSSI develops when individuals arise suicidal ideation is also a concern. According to the experiential avoidance model (EAM) of NSSI, in order to escape from unpleasant emotional experiences, individuals engage in NSSI and strengthen this behavior (42, 43). Specifically, when individuals faced with a similar situation again, NSSI would be an automatic escape reaction (44). Adolescents with NSSI have undeniable defects in emotional regulation, and they may experience more negative emotions (45). Suicidal ideation is often inseparable from certain negative emotions (depression, hopelessness, anxiety, etc.) (46–48). When strong suicidal ideation cannot be dispelled, NSSI may be consequence for adolescents to reduce negative effects. Thus, this study hypothesized that current suicidal ideation would predict subsequent NSSI.

In summary, the purposes of this study are to answer three questions: (1) whether there is a relationship between suicidal ideation and NSSI at any point in time; (2) whether NSSI would predicts later suicidal ideation; and (3) whether current suicidal ideation predicted subsequent NSSI.

## Materials and methods

### Participants and procedure

For the current study, we conducted a prospective longitudinal study of adolescents from a middle school in Tianjin, China through three measurements. The first measurement was conducted in the fourth week of the second semester from 2016 to 2017 academic year.

Data were collected as part of a school-wide psychological assessment of a middle school. All procedures and ethical aspects of this study were approved by ethics committee of the Academy of Psychology and Behavior of Tianjin Normal University (XI2020–03) and the middle school. To avoid discomfort of participants, all questions about suicidal ideation and NSSI behaviors were intermixed with other items. The on-site hosts and supervisors were some postgraduate psychology students who had received specific training in advance. Before filling in the questionnaires, participants were given a brief description of the study and told that the answers would be confidential. In principle of voluntary participation, all participants also filled out informed consent forms for their participation. And they were given a small gift in return for completing the questionnaire.

### Measures

#### Demographics

Participants completed a demographic questionnaire that assessed age, sex, family economic status (compared to the local average level), one-child in family, household type, national, regions.

#### Suicide ideation

One item of the Suicide ideation/suicide attempt questionnaire were drawn for measuring how often adolescents thought about suicide in the past week. This item used a four-point scale (1 = occasionally or none; 2 = sometimes; 3 = often; 4 = duration), the higher the score, the stronger the suicidal ideation. Although the scale has only one item for measuring suicidal ideation, it is widely used in suicide research (49, 50). In this study, all items were in Chinese.

#### NSSI

NSSI was measured by the Adolescents Self-Harm Scale (ASHS), which was well used in Chinese research (51). We chose 3 items to assess adolescents’ self-injury from the scale, such as “ Have you ever intentionally cut, burned, slashed yourself, or hurt yourself in any other way?”, “Have you ever intentionally poked open a wound to stop it from healing?” and “Have you ever hurt yourself physically by intentionally letting someone else hit or bite you?” Participants needed to response every item in “ Yes “ or “No”. In this study, all items were in Chinese.

### Data analytic strategy

Firstly, descriptive analyses were examined including bivariate correlations between suicidal ideation and NSSI behaviors and demographic characteristics of participants in SPSS24.0. In addition, we use kurtosis and skewness to test if the data are normally distributed. It can be considered as a normal distribution when the two metrics are closer to zero.

Although suicide ideation has been associated with NSSI behaviors in previous studies, these studies have not established a causal relationship (i.e., whether NSSI behaviors is affected by suicide ideation, or whether suicide ideation is affected by NSSI behaviors). The relationship between two or more observational variables over time can be analyzed using an autoregressive cross-lag model. Therefore, this study constructed a three-wave cross-lagged model to clarify the issue with M-plus7.0.

According to the analysis procedure of van Lier et al. (52), we constructed four models. And we analyzed diffierences in global model fit between them to examine contributions longitudinally of each predictive path. The first model (M1) is a autoregressive model (baseline model) specifying no cross-lagged longitudinal effiects. The second model (M2) only contains the cross-lagged regression path from X (suicidal ideation) to Y (NSSI) in one direction, and the others are the same as M1. The third model (M3) only contains the cross-lagged regression path from Y (NSSI) to X (suicidal ideation) in one direction, and the others are the same as M1. The fourth model (M4) is the full model, which both includes the cross-lagged regression path from X to Y and from Y to X.

The model analysis adopted the maximum likelihood estimator with robust standard errors (MLR), which is suitable for non-normal distribution and non-independent data and can provide statistical indicators such as standard error and chi-square value for the non-normal data and process the missing data at the same time (53). Four criteria were used to evaluate the fit of the model (54, 55): the chi-square (χ^2^; less degrees of freedom suggests a good fit), the comparative fit index (CFI ≥ 0.95 suggests a good fit), the root-mean-square error of approximation (RMSEA < 0.08 suggests a good fit), and the standardized root-mean-square residual (SRMR ≤ 0.08 suggests a good fit).

## Results

### Descriptive statistics

A total of 293 paper-and-pencil version questionnaires were issued and 271 were effectively collected (valid response rate: 92.49%), including 140 boys and 131 girls. The second measurement was conducted in the fourth week of the first semester of the 2017–2018 academic year, and 231 people were tracked, with a tracking rate of 85.24%. Participants include 113 boys and 118 girls, 40 of whom were lost. The third measurement was conducted in the fourth week of the second semester of the 2017–2018 academic year, and 193 people were tracked, with a tracking rate of 83.55%. It includes 95 boys and 98 girls, 38 of whom were lost. The average age in this final sample was 14.45 years (SD = 0.61). Other demographic characteristics were outlined in **Table 1**.

**Table 1:** Demographic characteristics of sample.

	N	Percentage
Gender		
*Male*	*98*	49.20%
*Female*	*98*	50.80%
Family economic status (Compared to the local average level)		
Far above	1	0.50%
Slightly above	40	20.70%
Average level	130	67.40%
Slightly below	20	10.40%
Far below	2	1.00%
Household type		
The nuclear family	141	73.10%
Single parent families	8	4.10%
Others	44	22.80%
One-child in family		
Yes	62	32.10%
No	131	67.90%
National		
Han	188	97.40%
Manchu	2	1.00%
Others	3	1.60%
Regions		
urban	73	37.80%
rural	120	62.20%

The results of an independent sample t-test showed no significant difference in suicidal ideation and NSSI behaviors (*p* > 0.05) between subjects for whom data were excluded and retained, indicating that was random.

In addition, the results of skewness and kurtosis showed that not all variables at all-time points conform to normal distribution. Other descriptive characteristics were outlined in **Table 2**.

**Table 2:** Means, standard deviations, ranges, skewness, kurtosis.

	T1 SI	T2 SI	T3 SI	T1 NSSI	T2 NSSI	T3 NSSI
Percentage	25.90%	21.20%	28.00%	20.20%	16.10%	20.20%
M ± SD	1.45 ± 0.88	1.32 ± 0.70	1.46 ± 0.83	0.29 ± 0.65	0.23 ± 0.61	0.33 ± 0.72
Range	1–4	1–4	1–4	0–3	0–3	0–3
Kurtosis	1.99	2.44	1.68	2.5	2.96	2.25
Skewness	2.87	5.59	1.7	6.01	8.93	4.24

T1 SI, Time 1 Suicidal Ideation; T2 SI, Time 2 Suicidal Ideation; T3 SI, Time 3 Suicidal Ideation; T1 NSSI, Time 1 NonSuicidal Self-Injury Behaviors; T2 NSSI, Time 2 NonSuicidal Self-Injury Behaviors; T3 NSSI, Time 3 NonSuicidal Self-Injury Behavior.

### Correlation analysis of variables

Bivariate correlations between suicidal ideation and NSSI behaviors were presented in **Table 3**. The correlation of suicidal ideation between time points T1, T2 and T3 was significant, which the correlation coefficient was 0.30 ~ 0.50; There was also a significant correlation between NSSI behaviors at time points T1, T2 and T3, with the correlation coefficient of 0.53 ~ 0.66. The above data show a certain stability of suicidal ideation and NSSI levels of adolescents. **Table 3** also showed a significant contemporaneity correlation between suicidal ideation and NSSI at time points T1, T2, and T3. At the same time, the stepwise correlation between them at T1, T2 and T3 was also significant.

**Table 3:** Correlations of study variables.

	T1 SI	T2 SI	T3 SI	T1 NSSI	T2 NSSI	T3 NSSI
T1 SI	–					
T2 SI	0.30^***^	–				
T3 SI	0.28^***^	0.50^***^	–			
T1 NSSI	0.39^***^	0.29^***^	0.24^***^	–		
T2 NSSI	0.33^***^	0.27^***^	0.17^***^	0.63^***^	–	
T3 NSSI	0.23^***^	0.30^***^	0.26^***^	0.53^***^	0.66^***^	–

T1 SI, Time 1 Suicidal Ideation; T2 SI, Time 2 Suicidal Ideation; T3 SI, Time 3 Suicidal Ideation; T1 NSSI, Time 1 NonSuicidal Self-Injury Behaviors; T2 NSSI, Time 2 NonSuicidal Self-Injury Behaviors; T3 NSSI, Time 3 NonSuicidal Self-Injury Behaviors.

***p < 0.001, **p < 0.01, *p < 0.05, same as below.

### Cross-lagged model analysis of variables

In the environment of M-plus7.0, Cross-lagged analysis was used to explore the relationship between suicidal ideation and NSSI of adolescents. The model fitting information of each competition model (M1-M4) was shown in **Table 4**.

**Table 4:** Model fit indices of the three-wave panel model.

Model	χ^2^	*p*	df	CFI	TLI	RMSEA	SRMR	Δχ^2^	*p*	Δdf	Scaling Correction Factor
M1	22.021^**^	0.009	9	0.925	0.884	0.087	0.095	–	–	–	1.37
M2	15.751^*^	0.028	7	0.950	0.900	0.080	0.075	6.22^*^	0.045	2	1.36
M3	16.238^*^	0.023	7	0.947	0.894	0.083	0.060	5.81	0.055	2	1.38
M4	10.029	0.074	5	0.971	0.919	0.072	0.037	12.01^*^	0.017	4	1.37

χ^2^, Scaled Chi-Square; df, Degrees of Freedom; CFI, Comparative Fit Index; TLI, Tucker-Lewis Index; RMSEA, Root Mean Square Error of Approximation; SRMR, Standardized Root Mean Square Residual; Δχ^2^, Satorra-Bentler Chi-Square Difference Test. ***p < 0.001, **p < 0.01, *p < 0.05.

First, a baseline model (M1) was tested to estimate the stability coefficient of the relationship between suicidal ideation and NSSI in adolescents. The Cross-lagged paths of two variables at three time points were not examined. Baseline model (M1) fit indices was: χ^2^(9) = 22.021, *p* = 0.009, CFI = 0.925, RMSEA = 0.087, SRMR = 0.095. The model fit indices was not good and was not easy to be accepted.

Next, based on the baseline model, model 2 (M2: the cross-lagged regression path from suicidal ideation to NSSI in one direction) and model 3 (M3: the cross-lagged regression path from NSSI to suicidal ideation in one direction) are investigated respectively. Compared with M1, the results of these two models have improved but still indicated a lack of perfect fit: Δχ^2^_(M2-M1)_ = 6.22, Δdf_(M2-M1)_ = 2, *p* = 0.045; Δχ^2^_(M3-M1)_ = 5.81, Δdf_(M3-M1)_ = 2, *p* = 0.055.

Finally, stability and all cross-lagged path (M4) of that relationship between suicidal ideation and NSSI of adolescents were simultaneously detect. The results of M4 showed that the model fit was good: χ^2^(5) = 10.029, *p* = 0.074, CFI = 0.971, RMSEA = 0.072, SRMR = 0.037. Again, compared with M1,the results were Δχ^2^_(M4-M1)_ = 12.01, Δdf_(M4-M1)_ = 4, *p* = 0.017.This indicated that there are significant differences between two models, and M4 were the best one to reflect the relationship between variables among all the models. Therefore, model 4 served as the final model we use for analysis.

**Figure 1:**
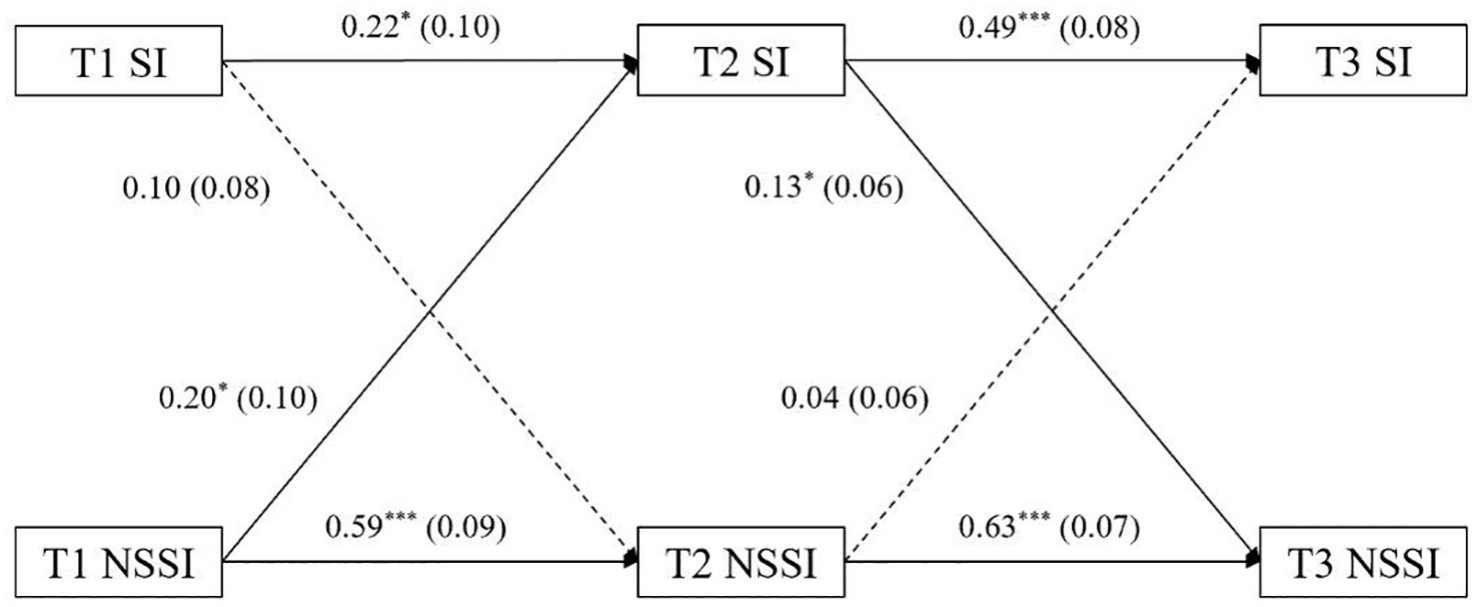
Graph of cross-lagged model of suicidal ideation and nonsuicidal self-injury. All coefficients are standardized regression coefficients (*β*); Behind the coefficient is standard error (*SE*); One-way arrow solid line indicates the effect is significant, dotted line indicates the effect is not significant. ***p < 0.001, **p < 0.01, *p < 0.05.

**Figure 1** presented the results of the cross-lagged analysis of the model 4. NSSI (0.59 ~ 0.63) was more stable than suicidal ideation (0.22 ~ 0.49). The NSSI at time T1 significantly positively predicted suicidal ideation at time T2. Suicidal ideation at time T2 also significantly positively predicted NSSI at T3.

## Discussion

### The stability of suicidal ideation and NSSI among adolescents

The bivariate correlation analysis showed that the correlation between suicidal ideation and NSSI was higher and higher at the three time points. This suggested that the levels of suicidal ideation and NSSI among adolescents are somewhat stable across time, in line with the differential activation theory of suicidality (DAT), which believed that suicidal ideation occurs repeatedly (56). In particular, the study found that individuals with depression also have an extremely high recurrence rate of suicidal ideation (57–59). In a longitudinal study of the epidemiology of suicidal ideation among urban and rural Chinese adolescents, age and suicidal ideation history were the only predictors of future suicidal ideation (60). According to the EAM of NSSI, it has the function of emotion management (42, 44). This meant that before NSSI, adolescents usually had strong negative experiences such as frustration, depression, helplessness and stress, while after NSSI, negative experiences are alleviated or eliminated, and the adolescents feeled relieved and relaxed (45, 61). This process was seen as a reinforcing effect, meaning that the same behavior will happen again in a similar situation (42, 62).

Therefore, it was not difficult to understand that the suicidal ideation and NSSI of adolescents will occur repeatedly when adolescents continuously face situations, such as academic pressure and unhealthy interpersonal relationships, that evoke negative emotions over a long period.

### NSSI as a gateway of suicidal ideation among adolescents

The results of the cross-lagged model analysis showed that NSSI at time T1 significantly positively predicted suicidal ideation at time T2. This indicated that the more NSSI adolescents experienced, the higher the possibility of suicidal ideation. Since adolescents are not mature, physically and mentally, they are more likely to be affected by a sense of perceived burdensomeness and thwarted belongingness (63, 64). However, these two feelings alone were not enough to make adolescents develop thoughts about suicide (35). Adolescents who engaged in NSSI were more likely to have suicidal thoughts than those who did not (23). In line with the opinion of IPTS, NSSI was a gateway to suicidal ideation in adolescents (31, 35). NSSI is caused not only by negative events, but also by adolescents’ curious and rebellious nature. Furthermore, repeated NSSI gave adolescents a higher pain tolerance and an acquired capability for suicide (by overcoming the internal fear) (65). As a result, NSSI is transformed into suicidal ideation.

### NSSI as a consequence of suicidal ideation among adolescents

Another result of the cross-lagged model analysis showed that suicidal ideation at time T2 also significantly positively predicted NSSI at timeT3. This suggested that NSSI was not only the cause of suicidal ideation, but also the result of suicidal ideation. It was unclear the effect of suicidal ideation on NSSI. In order to avoid the influence of negative emotions in suicidal ideation, adolescents adopt the way of NSSI to alleviate negative feelings according the EAM of NSSI (42, 45). However, the NSSI at this time has an inherent essential difference from the first one, and it is riskier. That’s another question. Why the NSSI at time T1 and suicidal ideation at time T2 significantly and positively predicted suicidal ideation at time T2 and NSSI at time T3 separately, but neither suicidal ideation at time T1and the NSSI at time T2 significantly and positively predicted NSSI and suicidal ideation at time T3 separately. Our study suggested that suicidal ideation and NSSI can predict each other in the longitudinal, but this relationship is a chronological. Briefly, a series of effects after this all depend on the first cause of NSSI. Therefore, strengthening the prevention and intervention of Chinese adolescents’ NSSI plays an extremely important role in the occurrence of suicidal ideation and behaviors (2, 23).

### Limitations

There are still several limitations should also be mentioned, though this study has some merit. First, the measurement tools we used are all simple questionnaires, which needs to be validated in future studies, and more complete measurement tools can be adopted in the future research. For example, we can consider the relationship between the frequency and pattern of NSSI and suicidal ideation. Second, our sample was one of convenience with Chinese adolescents, and it is not clear to what extent the results of our research are applicable to other Chinese people. In addition, given that the small number of participants with suicidal ideation and NSSI, the findings of the study should be interpreted with caution and a lager sample is needed to be collected and analyzed in the future. Thirdly, due to the inherently dynamic nature of suicide ideation (66, 67), although cross-lagged model can help us observe changes in suicidal ideation and self-injury, shorter observation times could be adopted in the future.

## Conclusions

In this study, NSSI and suicidal ideation over a long period of time among adolescents were analyzed together. Our study suggested that, if the occurrence and development of NSSI and suicidal ideation were regarded as a linear process, there was a reciprocally predictive relationship between them. NSSI may occur before suicidal ideation, which in turn would strengthen NSSI. This also prompted that interventions should be carried out from two aspects (behaviors and thoughts) to improve adolescents’ mental health.

## Data Availability

The datasets presented in this article are not readily available because of participant confidentiality and privacy. Requests to access the datasets should be directed to linlin@tjnu.edu.cn.
